# Decreasing temperature enhances the formation of sixfold hydrogen bonded rings in water-rich water-methanol mixtures

**DOI:** 10.1038/s41598-017-01095-7

**Published:** 2017-04-21

**Authors:** Imre Bakó, László Pusztai, László Temleitner

**Affiliations:** 1 Research Centre for Natural Sciences, Hungarian Academy of Sciences, H-1117 Budapest, Magyar tudósok körútja 2., Hungary; 2 Institute for Solid State Physics and Optics, Wigner Research Centre for Physics, Hungarian Academy of Sciences, H-1121 Budapest, Konkoly Thege út 29-33, Hungary

## Abstract

The evolution of the structure of liquid water-methanol mixtures as a function of temperature has been studied by molecular dynamics simulations, with a focus on hydrogen bonding. The combination of the OPLS-AA (all atom) potential model of methanol and the widely used SPC/E water model has provided excellent agreement with measured X-ray diffraction data over the temperature range between 298 and 213 K, for mixtures with methanol molar fractions of 0.2, 0.3 and 0.4. Hydrogen bonds (HB-s) have been identified via a combined geometric/energetic, as well as via a purely geometric definition. The number of recognizable hydrogen bonded ring structures in some cases doubles while lowering the temperature from 298 to 213 K; the number of sixfold rings increases most significantly. An evolution towards the structure of hexagonal ice, that contains only sixfold hydrogen bonded rings, has thus been detected on cooling water-methanol mixtures.

## Introduction

Despite the voluminous literature available on liquid methanol and methanol-water mixtures, the flow of new investigations on the subject is continous (see, e.g., refs 1–5). The reason, apart from the enormous industrial significance of methanol, is that the molecules of methyl-alcohol, CH_3_OH, are the closest organic analogues, by their size and shape, of water molecules, but they contain a hydrophobic methyl (CH_3_-) group. These ambivalent properties of methanol molecules give rise to a complex structure of its mixtures with water: hydrogen bonds (HB-s) may form between water–water and methanol–methanol, as well as between water–methanol and methanol–water pairs. Characteristics of hydrogen bonds in methanol and water-methanol mixtures have been considered by numerous publications^5–8^; for mixtures, these analyses have often been conducted as a function of composition^1, 2, 9^.

Interestingly, hydrogen bonding as a function of temperature has not yet been analysed in detail in water-methanol mixtures. One of the reasons may be the lack of suitable experimental data: while diffraction results for ambient conditions are abundant, both by X-rays^2, 10–12^ and by neutrons^6, 7^, to our best knowledge, only one X-ray data set is available for temperatures down to the freezing point^12^. This lack of information has turned our attention to structural investigations at lower temperatures.

In a recent work, some of the present authors reported a detailed analysis of molecular dynamics computer simulation data for the microscopic structure of water-methanol mixtures in the entire composition range and performed comparisons with the experimental results obtained by X-ray diffraction^2^. The principal focus in that publication was on the changes of the experimental and theoretical total structure factors of mixtures as a function of composition, considered at room temperature and ambient pressure. On the molecular dynamics (MD) computer simulation side, we analyzed the SPC/E^13^ and TIP4P-Ew^14^ water models, in combination with the OPLS-AA methanol model^15^.

Encouraged by an overall satisfactory performance of the molecular dynamics simulation data for describing experimental trends, here we take temperature dependent X-ray diffraction data of Takamuku *et al*.^12^ and perform molecular dynamics simulations for compositions and temperatures of the diffraction experiments, using the same interatomic potential functions (SPC/E for water and OPLS-AA for methanol) as previously^2^. The resulting particle configurations have been analyzed by the tools reported in refs 4 and 9 for identifying hydrogen bonds and find topological characteristics of their networks.

Ring structures composed by hydrogen bonded molecules are an easily recognizable type of hydrogen bonded assemblies. Their significance is indicated by the fact that the structure of one of best known material in everyday life, crystalline ice, is entirely built of rings that contain 6 hydrogen bonded water molecules^16^. Cyclic entities have previously been found in simulated water-methanol mixtures at room temperature over the entire composition range^9^, although the number of rings decreased rapidly as methanol concentration grew. In this work, the temperature evolution of the number of cyclic entities, that are kept together via hydrogen bonds, is followed. We expect that the insights provided by the present investigation will contribute significantly to our understanding of structural changes that occur in hydrogen bonded systems upon lowering the temperature.

## Results

### Total structure factors

**Figure 1:**
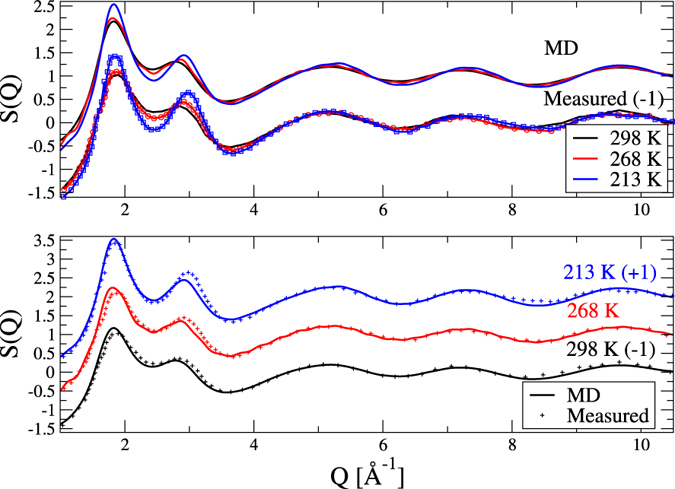
Temperature dependence of simulated and measured X-ray weighted total structure factors for the water-methanol mixture with a methanol molar ratio of 0.4. Upper panel: comparison of trends observed while lowering the temperature. Lower panel: individual comparisons of simulated and measured structure factors at three temperatures.

Figure 1 compares X-ray weighted total structure factors, *S*(*Q*), as measured by X-ray diffraction^12^ and as calculated from the present molecular dynamics computer simulations for the mixture with *Xm* = 0.4 (*Xm* is the molar fraction of methanol). The applied interatomic potentials have proven to be adequate for reproducing X-ray diffraction data over the temperature range between 298 and 213 K, and for methanol molar fractions of 0.2, 0.3 (not shown) and 0.4. Simulated curves follow the same trends with temperature as those obtained from diffraction experiments; moreover, calculated *S*(*Q*)-s match the corresponding measured functions at an at least semi-quantitative level. Deviations between experiment and simulation could only be observed around the second maximum of the total scattering structure factor, similarly to what had been found for room temperature diffraction data^2^. The very good agreement between diffraction data and simulated total scattering structure factors provides a strong basis for detailed structural analyses. In other words, the present calculations may be considered as validated from the structural point of view, on the basis of the agreement with X-ray diffraction experiments.

### Radial distribution functions

**Figure 2:**
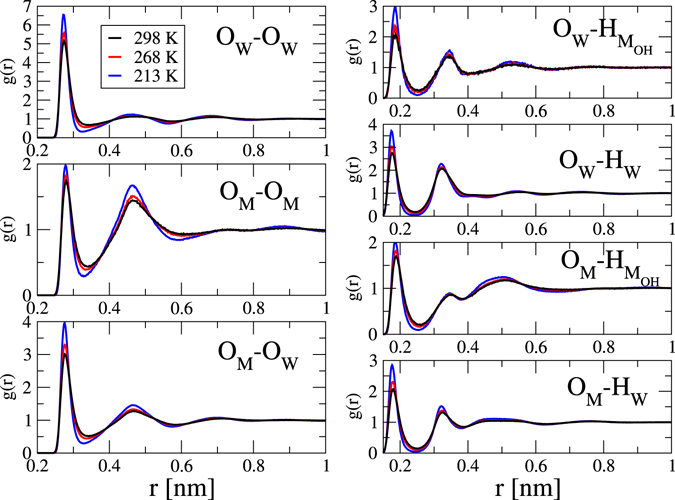
Temperature dependence of computer simulated partial radial distribution functions of water-methanol mixture with a methanol molar ratio of 0.4. Only the PRDF-s that are related to hydrogen bonding are shown. Changes with temperature are systematic (although hardly visible).

Partial radial distribution functions, PRDF, are inaccessible from diffraction experiments for water-methanol mixtures and therefore, only computer simulation results may be presented in Fig. 2. Possessing atomic coordinates, it would be possible to calculate all the PRDF-s; here we restrict ourselves to displaying the functions that are related to hydrogen bonding, i.e., all possible combinations of O-O and O-H partials. Each of the 7 PRDF-s show the same trends as temperature decreases: maxima and minima become slightly, but systematically sharper. Also, slight shifts in terms of the positions of first O-O maxima and minima can be observed, due to the slight change of the density with decreasing temperature. What is important to put on the record at this stage is that the observed changes in terms of two-body correlations (the PRDF-s) are consistent with common expectations and that these changes are small. These findings are in qualitative agreement with the suggestions of Takamuku *et al*.^12^.

### Hydrogen bond characteristics

Hydrogen bonds have been identified both by energetic and/or geometric considerations, as in previous publications^2, 4, 9^. According to these preliminaries, two molecules are identified as H-bonded if the non-bonding distance between an oxygen and a hydrogen atom is less than 2.5 Å, and (a) the O…O–H angle is smaller than 30 degrees (geometric definition), or (b) the interaction energy between the H-bonded molecules is less than −3.0 kcal/mol (energetic definition). All calculations concerning H-bonding and the H-bonded network have been performed by the HBTOPOLOGY code, originally developed by Chihaia *et al*.^17^.

Based on Table 1 the following observations can be made:

(i) The number of energetically defined hydrogen bonds is lower, by about 5 to 10%, than the number of hydrogen bonds identified via purely geometrical means. This difference is roughly constant over the entire composition and temperature range considered here, so that all the trends that may be identified for one definition is valid for the other one, as well. Nevertheless, key quantities will be mentioned for both definitions, so that it is ensured that the main findings are not influenced by the H-bond definition.
(ii) The change in terms of the number of hydrogen bonds is almost negligible over the last 10 K of the temperature range covered. Nevertheless, in what follows, results for both LT1 and LT2 will be shown since LT2 for *X*_*m*_ = 0.2 and 0.3 falls in the supercooled regime. On the other hand, LT1 is safely within the normal liquid range for each composition considered.
(iii) The average number of hydrogen bonds per molecule increases by about 10% as temperature decreases from room temperature by 55 (*X*_*m*_ = 0.2) to 85 (*X*_*m*_ = 0.4) K.
(iv) The increment in terms of the number of H-bonded molecules is greater around water molecules than around methanol molecules.
(v) In contrast to the number of H-bonds, the number of cyclic entities per molecule increases by between 60 (*X*_*m*_ = 0.2) and 100 (*X*_*m*_ = 0.4) % when temperatures drop down to LT2 (concerning LT1, the increase is between 50 and 90%, respectively).

**Table 1:** Hydrogen-bond statistics in the systems studied, as calculated for the energetic definition of hydrogen bonds (values in parantheses correspond to the geometric definition of H-bonds).

*X*_*m*_		0.4	0.3	0.2
N_*hb*_^*a*^	298 K^*g*^	2.67 (2.88)	2.82 (3.05)	2.95 (3.23)
	268 K^*h*^	2.79 (2.99)	2.95 (3.17)	3.10 (3.35)
	LT1^*i*^	2.96 (3.11)	3.10 (3.28)	3.17 (3.41)
	LT2^*j*^	2.99 (3.13)	3.14 (3.31)	3.21 (3.44)
*N*_*ww*_^*b*^	298 K^*g*^	2.17 (2.37)	2.45 (2.69)	2.71 (2.99)
	268 K^*h*^	2.27 (2.45)	2.56 (2.78)	2.84 (3.10)
	LT1^*i*^	2.40 (2.54)	2.69 (2.87)	2.91 (3.15)
	LT2^*j*^	2.42 (2.55)	2.73 (2.89)	2.95 (3.18)
*N*_*mm*_^*c*^	298 K^*g*^	0.56 (0.62)	0.42 (0.47)	0.28 (0.31)
	268 K^*h*^	0.58 (0.63)	0.43 (0.47)	0.28 (0.31)
	LT1^*i*^	0.59 (0.63)	0.44 (0.47)	0.29 (0.31)
	LT2^*j*^	0.59 (0.63)	0.44 (0.47)	0.29 (0.31)
*N*_*mw*_^*d*^	298 K^*g*^	1.42 (1.51)	1.63 (1.72)	1.83 (1.94)
	268 K^*h*^	1.50 (1.58)	1.71 (1.80)	1.92 (2.03)
	LT1^*i*^	1.60 (1.66)	1.80 (1.88)	1.96 (2.06)
	LT2^*j*^	1.63 (1.69)	1.82 (1.89)	1.99 (2.09)
*N*_*wm*_^*e*^	298 K^*g*^	0.95 (1.00)	0.70 (0.74)	0.46 (0.49)
	268 K^*h*^	1.00 (1.06)	0.73 (0.77)	0.48 (0.50)
	LT1^*i*^	1.07 (1.11)	0.77 (0.80)	0.49 (0.51)
	LT2^*j*^	1.09 (1.13)	0.78 (0.81)	0.50 (0.52)
*N*_*c*_/*N*_*o*_^*f*^	298 K^*g*^	0.27 (0.43)	0.38 (0.62)	0.51 (0.82)
	268 K^*h*^	0.35 (0.53)	0.50 (0.74)	0.66 (0.98)
	LT1^*i*^	0.51 (0.66)	0.66 (0.80)	0.75 (1.06)
	LT2^*j*^	0.53 (0.68)	0.71 (0.91)	0.81 (1.11)

^*a*^Average number of hydrogen bonds per molecule; ^*b*^average number of H-bonded water molecules around water; ^*c*^average number of H-bonded methanol molecules around methanol; ^*d*^average number of H-bonded water molecules around methanol; ^*e*^average number of H-bonded methanol molecules around water; ^*f*^average number of cyclic entities per molecule; ^*g*^actual temperature over freezing point ratio (T/T_*f*_): 1.40, 1.31, 1.21; ^*h*^actual temperature over freezing point ratio (T/T_*f*_): 1.26, 1.17, 1.09; ^*i*^corresponds to 223 K, 233 K and 253 K, respectively, with T/T_*f*_: 1.05, 1.02, 1.03; ^*j*^corresponds to 213 K, 223 K and 243 K, respectively, with T/T_*f*_: 1.00(5), 0.98, 0.99.

The (huge) extent of the increase in terms of the number of cyclic entities would be impossible to predict on the sole basis of points (i) to (iv); it is therefore thought that this finding may termed as ‘surprising’ and ‘unexpected’. In contrast, data concerning the number of H-bonds in Table 1 fits well into the general views on what happens in H-bonded systems on cooling, in accordance with partial radial distribution functions (see Fig. 2) and with suggestions by Takamuku *et al*. reported in their experimental study^12^.

**Figure 3:**
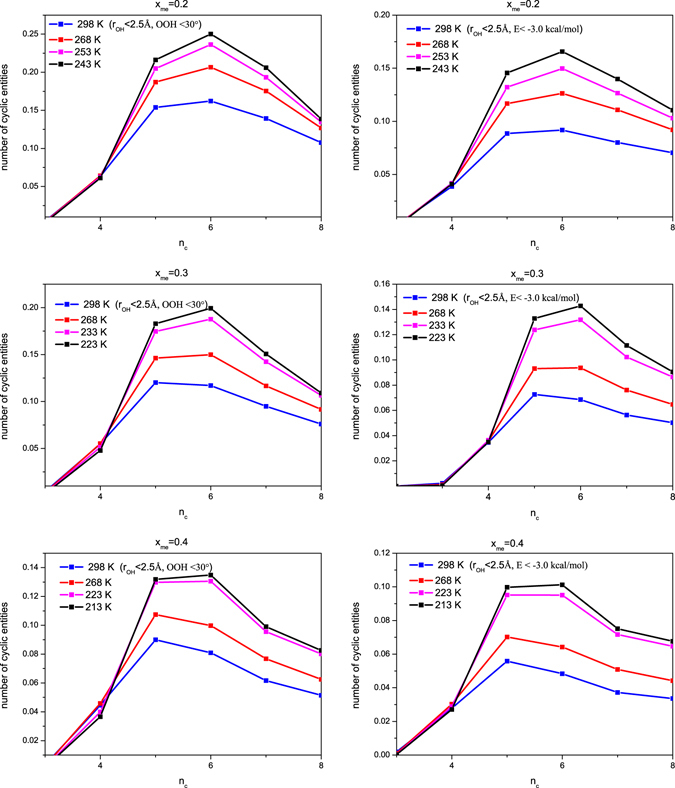
Ring size distributions, normalized by the number of molecules in the simulated systems, for the three mixtures (upper panels: X_*M*_ = 0.2; middle panels: X_*M*_ = 0.3; lower panels: X_*M*_ = 0.4), as a function of temperature. Left panels: geometric definition of hydrogen bonds; right panels: energetic definition. Sixfold rings become the most abundant at the lowest temperature (LT2) at each methanol content; also, their abundance nearly doubles at the lowest temperatures in comparison with room temperature values.

The last finding of shown in the above list [‘(*v*)’] led us to look at primitive cyclic entities (the ones that cannot be split into smaller rings^17^) more closely. Figure 3 displays ring size distributions, as a function of temperature, for the mixtures simulated in the present study, for H-bonded cycles containing up to 8 molecules.

The first thing to notice is that for both definitions of H-bonds and for each composition the overall number of cycles becomes progressively larger as the temperature approaches the experimental freezing temperature. The increase is significant for 5-, 6- and 7-membered rings, and it is outstanding for the 6-membered cycles. At higher temperatures and higher methanol contents, 5 membered rings are slightly more abundant than 6-membered ones, but at the lowest temperature, 6-membered primitive cycles become dominant at each concentration. The composition of 6-membered rings is, apparently, not sensitive to decreasing the temperature: for instance, for the X_*m*_ = 0.2 mixture at 298, as well as at 243 K, 58% of such cyclic entities contain no methanol molecule at all, whereas 1 and 2 methanol molecules can be found in 36 and 5%, respectively, of the 6-membered rings. The compositions of 5- and 7-membered cycles are similarly stable against temperature.

The fact that 6-membered rings contain significantly more water molecules than it would follow from the composition of the mixtures indicates that these homogeneous systems look microscopically somewhat heterogeneous, particularly as temperature goes down – it might even be said that one component (6-membered rings of water molecules) is dispersed in a homogeneous ‘matrix’. This point would certainly be important to investigate for mixtures with higher alcohol contents.

**Figure 4:**
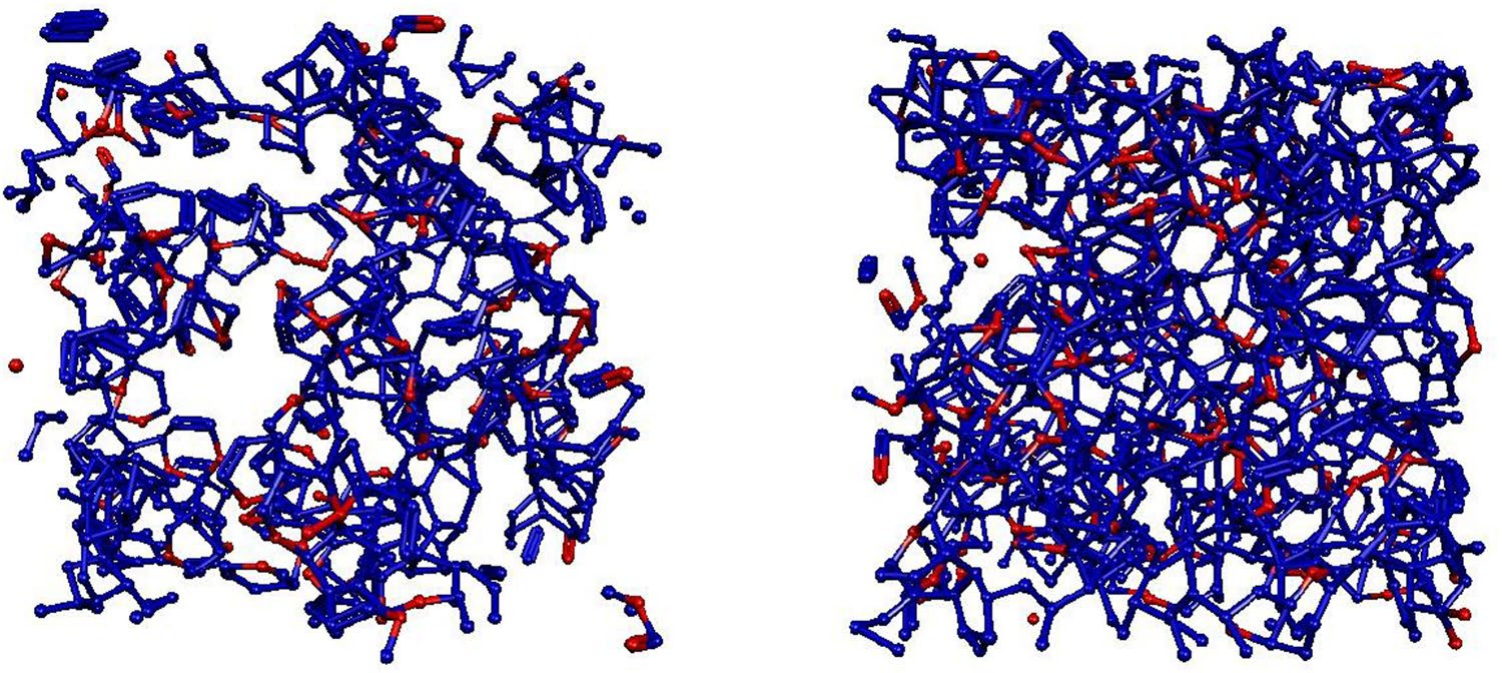
Sixfold rings in the mixture with X_*m*_ = 0.2 at room temperature (left panel) and close to the freezing point (right panel). Blue: water molecules; red: methanol molecules.

In order to give an idea about the populations of 6-membered rings in the X_*m*_ = 0.2 mixture at 298 and at 243 K, Fig. 4 draws these cycles at these two temperatures (these drawings are taken from typical single particle configurations, using the energetic definition of H-bonds; note that for the geometric definition the number of sixfold rings is even larger). While the presence of 6-membered rings at room temperature is already significant, they seem to fill the entire simulation box at the lowest temperature considered (243 K). The change is dramatic; we make an attempt of providing a simple, qualitative explanation in the next section.

## Discussion

In the light of the above findings, it is rather unfortunate that experimental data are available for only these compositions, all in the water-rich regime. This is the reason why we are unable to extend experimental data based investigations to methanol-rich compositions. For pure water (0 molar % methanol) it is known^18, 19^ that the SPC/E potential model cannot reproduce diffraction data at room temperature; for this reason, pure water had to be left out from this study.

To the best of our knowledge, such greatly enhanced ring formation in water-alcohol mixtures on cooling has not been spotted in simulated structures that were in good (i.e., at least, semi-quantitative) agreement with measured diffraction data. For instance, the original paper of Takamuku *et al*.^12^ suggests that ‘alcohol-water mixtures are frozen with keeping the structure of the dominant clusters formed in the mixtures at 25 °C’, which clearly is different from what has been found here. The extent of the increase in terms of the number of H-bonded rings would be impossible to predict on the sole basis of the increase of H-bonds. Based on the present (although admittedly, limited) sample it may be conjectured that this is an essential phenomenon that may well be worth monitoring in other hydrogen-bonded systems, as well.

We suggest that a handwaving, qualitative explanation for the striking increase of cyclic entities could be that in hydrogen-bonded systems with substantial amounts of water, joining ‘dangling’ hydrogen bonds is an effective way of decreasing the entropy of the system. The presence of water is thought to be important since in all the known crystalline forms of water, H-bonded rings dominate; for instance, the most common form of crystalline water, ice Ih, consists of 6-membered rings exclusively. As pointed out in the previous section, primitive cycles identified in our simulated structures contain mostly water, with only about one methanol molecule per 6-membered ring on average. The clear domination of 6-membered rings in each low temperature particle configuration suggests that the appearance of a large number of cyclic entities is, while certainly lowering the configurational entropy of the system, likely to be a precursor of freezing.

As a final thought, it may be concluded that even though the enhanced ring formation might have looked extraordinary at first sight, it is quite easy to find ordinary, simple reasons for why it should happen in water-dominated alcohol-water mixtures. This is why it is interesting why this phenomenon has not been spotted earlier; one reason is a lack of suitable diffraction results to compare with – and it would be a desirable line of investigations to extend our knowledge base in this respect. Studying pure water in a similar fashion would be imperative, in order to check what happens in the pure substance that is known to form 6-membered rings in its common crystalline phase ice Ih. In parallel, it would be intriguing to explore the structure of pure alcohols as a function of temperature, since their crystalline phases do not consist of rings of hydrogen bonded molecules.

## Methods

All the simulations of water-methanol model mixtures in this work were performed by using the GROMACS molecular dynamics package^20^ (version 5.1.1) in the canonical ensemble at each temperature (298 down to 213 K) and at ambient pressure.

We chose the SPC/E^13^ model for water, combined with the OPLS-AA model^15^ for methanol, as this combination was shown to work well for methanol-water mixtures at room temperature^2^. The geometric combination rules, ${\varepsilon }_{ij}={({\varepsilon }_{ii}{\varepsilon }_{jj})}^{\mathrm{1/2}}$ and ${\sigma }_{ij}={({\sigma }_{ii}{\sigma }_{jj})}^{\mathrm{1/2}}$ for the interaction between unlike species (cross, non-bonded interactions) were used, together with the same rules for pure components. Non-bonded interactions were cut-off at 11 Å. In all the cases, bonds within methanol molecules were maintained fixed, whereas the angles and torsional angles were flexible. For the sake of maintaining the geometry of the water molecules the LINCS algorithm^21^ was used. The Particle Mesh Ewald method was used for treating Coulomb interactions^22^; the cutoff was again 11 Å.

Initially, particles were placed in a cubic simulation box randomly. Periodic boundary conditions were used. The number of molecules of both species in the simulation box for the three compositions is given in Table 2. Berendsen thermostat with *τ*_*T*_ = 0.1 ps was applied^23^; the timestep was 0.002 ps. Temperatures and densities were set according to the experimental conditions^12^; results are shown here for T = 298, 268, 253 and 243 K (*X*_*m*_ = 0.2), T = 298, 268, 233 and 223 K (*X*_*m*_ = 0.3), and T = 298, 268, 223 and 213 K (*X*_*m*_ = 0.4).

**Table 2:** Number of molecules of water and methanol in MD simulations, molar fraction of methanol and weight concentration.

*N*_*w*_	*N*_*m*_	*X*_*m*_ (%)	wt *m* (%)
1700	425	0.20	31
1400	600	0.30	43
1100	733	0.40	54

After equilibration, production runs were conducted. Simulations last 10 ns after equilibration. For hydrogen bond analyses, samples of 101 particle configurations, 10 ps apart, were collected.

For the identification of cyclic entities, the computer programme ‘HBTOPOLOGY’^17^ was applied; further applications of this code can be found in refs 4 and 9. We note that quite a few publications report on algorithms that can be used for the same purpose (see, e.g., refs 24–27 and references therein); HBTOPOLOGY was chosen since the source code was kindly made available to one of the present authors (I.B.) and therefore the transferability between various hydrogen bonded systems could be ensured.

The algorithm starts with setting up the connectivity list, according to the actual definition of hydrogen bonds. Each oxygen atom is taken as a ‘node’ in the network. The ring search is carried out on the basis of the connectivity of the nodes, considering one after another each node as starting-node. For a cyclic entity, the starting node has to be identical to the end node. The programme simply explores all possible paths that return to the starting node while touching a limited maximum number of nodes along the path. This pre-defined number was 6 in the original publication^17^; in the present case, cycles of up to 8 nodes have been searched for. Results presented in Fig. 3 concern primitive rings, i.e. ones that cannot be decomposed into smaller rings.
